# Prediction of components of the sporopollenin synthesis pathway in peach by genomic and expression analyses

**DOI:** 10.1186/1471-2164-14-40

**Published:** 2013-01-18

**Authors:** Gabino Ríos, Francisco R Tadeo, Carmen Leida, María L Badenes

**Affiliations:** 1 Instituto Valenciano de Investigaciones Agrarias (IVIA), Carretera Moncada-Náquera km 4.5, Moncada, Valencia, E-46113, Spain

## Abstract

**Background:**

The outer cell wall of the pollen grain (exine) is an extremely resistant structure containing sporopollenin, a mixed polymer made up of fatty acids and phenolic compounds. The synthesis of sporopollenin in the tapetal cells and its proper deposition on the pollen surface are essential for the development of viable pollen. The beginning of microsporogenesis and pollen maturation in perennial plants from temperate climates, such as peach, is conditioned by the duration of flower bud dormancy. In order to identify putative genes involved in these processes, we analyzed the results of previous genomic experiments studying the dormancy-dependent gene expression in different peach cultivars.

**Results:**

The expression of 50 genes induced in flower buds after the endodormancy period (flower-bud late genes) was compared in ten cultivars of peach with different dormancy behaviour. We found two co-expression clusters enriched in putative orthologs of sporopollenin synthesis and deposition factors in *Arabidopsis*. Flower-bud late genes were transiently expressed in anthers coincidently with microsporogenesis and pollen maturation processes. We postulated the participation of some flower-bud late genes in the sporopollenin synthesis pathway and the transcriptional regulation of late anther development in peach.

**Conclusions:**

Peach and the model plant *Arabidopsis thaliana* show multiple elements in common within the essential sporopollenin synthesis pathway and gene expression regulatory mechanisms affecting anther development. The transcriptomic analysis of dormancy-released flower buds proved to be an efficient procedure for the identification of anther and pollen development genes in perennial plants showing seasonal dormancy.

## Background

Sexual reproduction in angiosperms involves the formation of complex reproductive organs (flowers) containing diploid tissues and the haploid germline. The germline gives rise to the male (pollen) and female gametophyte (embryo sac) through successive meiotic and mitotic cell divisions from their respective microspore and megaspore mother cells. The genetic and molecular regulation of these events has been extensively studied in the model species *Arabidopsis thaliana*[1-3]. Pollen development and maturation occurs within the anther locule, surrounded by a specialized layer of helper cells named the tapetum. Tapetal cells greatly contribute to pollen viability and function through the segregation and deposition of the outer cell wall layer (exine) and the pollen coat (tryphine) on the pollen surface. The exine is an extremely durable and biochemically resistant structure consisting of sporopollenin, a series of complex polymers derived from fatty acids and phenolic compounds; whereas tryphine contains a sticky mixture of fatty acids, flavonoids, carotenoids and proteins deposited on the exine surface and cavities when the tapetum degenerates through programmed cell death [4,5].

Recently, several biochemical steps of sporopollenin biosynthesis and transcriptional regulatory circuits controlling pollen development have been elucidated in *Arabidopsis* by the analysis of male-sterile and exine-defective mutants [6]. In brief, medium- to long-chain fatty acids such as lauric acid are monohydroxylated by the cytochrome P450 CYP703A2 [7], and modified to form fatty acyl-CoA esters by ACYL-COA SYNTHETASE5 (ACOS5) in tapetal cells [8]. CoA-esterified fatty acids are alternatively reduced to form fatty alcohol derivatives or condensed with malonyl-CoA by LESS ADHESIVE POLLEN5/POLYKETIDE SYNTHASE B (LAP5/PKSB) and LAP6/PKSA, leading to alkyl pyrones [9,10]. These latter compounds are hydroxylated by TETRAKETIDE α-PYRONE REDUCTASE1 (TKPR1) and TKPR2 [11], and combined with phenylpropanoids to produce the sporopollenin precursors. Then sporopollenin is successively secreted to the apoplast by specific transporters [12] and translocated to the microspores bound to proteins such as lipid transfer proteins (LTPs) and glycine rich proteins (GRPs) [6]. A network of transcription factors containing basic helix-loop-helix (bHLH), plant homeodomain (PHD) finger, and MYB domains among others are likely regulating the expression of genes involved in these processes in the tapetum [13-18].

The knowledge regarding tapetum and pollen development in species other than the model organisms such as *Arabidopsis* and rice is scarce and fragmentary; in spite of the relevant influence that these processes exert on pollen viability, fruit set and productivity. Within the genus *Prunus*, including stone-fruit species as peach, plum, apricot, almond and cherry, several agronomical reports describe male-sterile varieties at the morphological and histological level [19-22]. However a consistent genetic and genomic analysis of processes affecting pollen viability is currently non-existent. The pollen development in *Prunus* species and other woody perennial plants from temperate climates such as apple and poplar is affected by the seasonal cessation of meristem growth termed endodormancy. Endodormancy contributes to elude the detrimental effects of the low temperatures in winter by preventing the resumption of growth under non-optimal conditions for survival. The growth inhibition of endodormant buds is due to internal signals within the buds, in contrast to growth inhibition by other distal organs (paradormancy), or by environmental factors (ecodormancy). For the purpose of this work we have employed the term dormancy to refer to the endodormant state. In these species, the flower buds start to differentiate in summer and continue their reproductive development until growth is arrested in autumn. After a period of chilling accumulation required for dormancy release, pollen mother cells within the anthers initiate meiosis and further microspore development, resulting in fully mature pollen grains [23].

In order to identify putative genes involved in tapetum function, pollen development and pollen wall formation in peach (*Prunus persica* [L.] Batsch), we analyzed the results of two transcriptomic experiments comparing gene expression between dormant and dormancy-released flower buds, and in peach cultivars with different dormancy behaviour [24,25]. This work led us to postulate a role for several genes in sporopollenin synthesis and deposition, and transcriptional regulation of pollen development processes, based on expression analysis and previous works in model species.

## Results and discussion

### Identification of genes up-regulated in late stages of reproductive bud development (flower-bud late genes)

**Figure 1:**
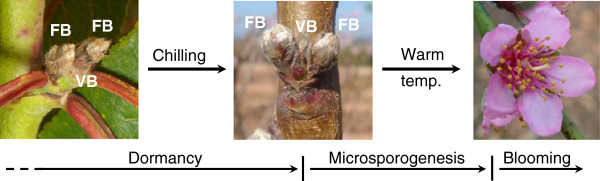
**Seasonal changes in flower development in peach.** Dormant buds (on the left) require a period of chilling to become competent for growing (on the middle). After dormancy, microsporogenesis is initiated, followed by flower opening after a period of warm temperatures (on the right). A single vegetative bud (VB) is typically flanked by two flower buds (FB).

Meristems of woody perennials from temperate climates go through the cold season in a dormant stage, protected into specialized structures named buds. In peach, reproductive (or flower) buds are typically arranged in pairs, flanking a single vegetative bud (Figure 1). In successive steps, flower buds are induced and differentiate in summer, and enter a dormancy period in autumn-winter. The dormancy is released after a required chilling period, whose length is genotype-specific. Finally their reproductive organs resume growth and development leading to blooming when temperature conditions become favourable (Figure 1). In anthers, the release of dormancy initiates microsporogenesis, pollen development and maturation [23].

We previously studied the genome-wide modification of gene expression in flower buds of peach through two complementary transcriptomic approaches. In the first work we isolated differentially enriched transcripts in dormant buds and dormancy-released buds by the suppression subtractive hybridization (SSH) procedure [24]. SSH procedure relies on the selective amplification and enrichment of abundant cDNAs in a sample (tester) when incubated and hybridized with an excess of a reference sample (driver). In the latter work cDNAs isolated by SSH were printed in a glass-microarray and used as probes in a microarray hybridization experiment against mRNA samples extracted from flower buds of ten peach cultivars with different chilling requirements for dormancy release [25]. Genes found to be up-regulated in flower buds during the dormancy transition, after the respective statistical analyses of SSH and microarray hybridization approaches are operationally termed in this work flower-bud late genes (Table 1). Most of these flower-bud late genes are described by transcript models predicted by the International Peach Genome Initiative (IPGI, http://www.rosaceae.org/peach/genome/), but nine lack a transcript profile, and consequently are designated by the unigene or EST name described in previous articles [24,25]. Three genes coding for putative peroxidases and LTP proteins were described by more than 40 ESTs each, which suggests a pronounced up-regulation of them under our experimental conditions.

**Table 1:** Flower-bud late genes obtained in transcriptomic studies in peach

**Transcript model**	**Blastx similarity**	**Number of ESTs**	Putative ortholog in Arabidopsis*	Reference*
ppa020321m	Peroxidase	74	At1g44970	[26]
ppa020886m	Protease inhibitor/seed storage/LTP	51		
ppa008309m	Peroxidase	45		
ppa003797m	4-coumarate-CoA ligase	19	*ACOS5*	[8]
ppa006506m	Strictosidine synthase	12	*LAP3*	[27]
ppa018509m	Protease inhibitor/seed storage/LTP	10	At3g52130	[26]
PpB87	Glycine-rich protein	9		
ppa017856m	Carboxyl-terminal peptidase	6		
ppa008548m	Cinnamoyl-CoA reductase	6	*TKPR1/DRL1*	[11]
ppa019432m	Chalcone synthase	5	*LAP6/PKSA*	[9,10]
ppa021373m	Protease inhibitor/seed storage/LTP	4		
ppa006739m	BURP domain protein	4		
ppa005767m	BURP domain protein	4		
ppa005535m	BURP domain protein	4		
PpB89	Glycine-rich protein	3		
PpB88		3		
PpB71	DNA-binding protein (AT-hook)	3	At2g42940	[28]
ppa025857m	Protease inhibitor/seed storage/LTP	3		
PpB79	Cupin	3		
ppa014645m	Carboxyl-terminal peptidase	3		
ppa009110m	Chlorophyll A-B binding protein	3		
ppa008777m	Cinnamoyl-CoA reductase	3	*TKPR2/CCRL6*	[11]
ppa005633m	Tubulin	3		
IB153		3		
ppa024968m		2		
ppa021109m	Protease inhibitor/seed storage/LTP	2		
ppa011965m	Short-chain dehydrogenase/reductase	2	*ATA1/TAPETUM1*	[13,29]
ppa009789m	MtN3/saliva family	2	*RPG1*	[30]
ppa006852m	Chalcone synthase	2	*LAP5/PKSB*	[9,10]
PpB91		1		
PpB60	Glucose-methanol-choline oxidoreductase	1		
ppb012876m		1		
ppa025137m		1		
ppa023338m	Plastocyanin-like	1		
ppa022178m	PHD Zn-finger protein	1	*MS1*	[15,16,31]
ppa020936m	BURP domain protein	1		
ppa016810m	Cytochrome P450	1	*CYP703A2*	[7]
ppa013829m	MazG nucleotide pyrophosphohydrolase	1		
ppa013711m	Early nodulin 93 ENOD93 protein	1		
ppa012800m	Protein of unknown function (DUF538)	1		
ppa011974m	Plastocyanin-like	1		
ppa010924m	Microsomal signal peptidase subunit	1		
ppa009350m	Xyloglucan endo-transglycosylase	1		
ppa008351m	Helix-loop-helix DNA-binding domain	1	*AtbHLH91*	[13,17]
ppa005764m	XYPPX repeat protein	1		
ppa005503m	Ubiquitin-like protein	1		
ppa004872m	Protein of unknown function (DUF668)	1		
ppa003411m	Multicopper oxidase	1		
ppa003039m	ATPase family (AAA)	1		
IB126		1		

* *Arabidopsis* genes related to sporopollenin and tapetum development that are putative orthologs of peach genes listed in this table (plus *RPG1*) are highlighted with their names and references.

Flower-bud late genes are expected to play a role in dormancy release, growth resumption or late flowering events. Whereas *DORMANCY ASSOCIATED MADS-box* (*DAM*) and other genes found repressed in dormancy-released buds have been unequivocally related to dormancy processes [32,33], no experimental evidences have been obtained pointing to a role of flower-bud late genes described in this work in dormancy processes. In order to identify putative orthologs of these genes in *Arabidopsis* we made a reciprocal blast analysis (RBA) as described in Methods. Interestingly, 13 genes were putative orthologs of *Arabidopsis* genes involved in sporopollenin synthesis and transcriptional regulation of tapetum and pollen development (Table 1, Additional file 1). In addition, ppa009789m was very similar to *RUPTURED POLLEN GRAIN1* (*RPG1*), a component of the MtN3/saliva gene family coding for a plasma membrane protein essential for microspore viability and exine pattern formation in *Arabidopsis*[30], even though they could not be considered as putative orthologs by RBA (Table 1). These data strongly suggest that flower-bud late genes identified by two transcriptomic approaches in peach [24,25] are to a large extent involved in sporopollenin synthesis and deposition, indicating the activation of this metabolic pathway during or shortly after dormancy release. Such predominance of pollen cell wall related genes over other bud processes, as dormancy release, abiotic stress resistance and female gametophyte development, could be due to the major contribution of anthers to the total weight of the bud, or alternatively could be caused by an experimental bias of the SSH procedure towards transcripts with higher expression differences.

### Flower-bud late genes show cultivar-dependent expression

**Figure 2:**
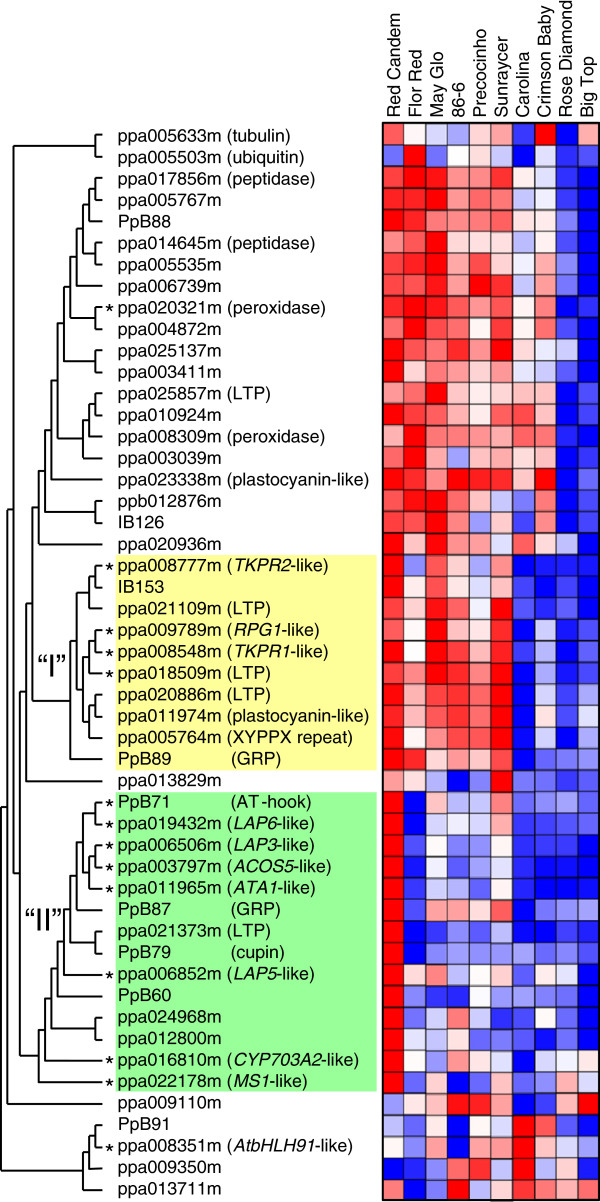
**Clustering of expression data from flower-bud late genes.** Genes are clustered according to their expression in flower buds of ten different cultivars (in columns) at a single collection time after 400 chilling hours. Asterisks label the putative orthologs of sporopollenin-biosynthesis and anther transcriptional factors of *Arabidopsis*. Clusters enriched in asterisk-labelled genes are coloured in yellow (I) and green (II). Microarray hybridization signals are represented by cells with a colour gradient from red (higher expression) to blue (lower expression).

The expression of ESTs from the 50 flower-bud late genes listed in Table 1 was extracted from microarray hybridization data stored in ArrayExpress database ( http://www.ebi.ac.uk/arrayexpress/) with accession number E-MEXP-3201. These expression data corresponded to flower buds from ten different peach cultivars collected the same day, after the accumulation of 400 chilling hours (hours below 7°C), a time approximately intermediate between the chilling requirements of earlier and later cultivars [25]. A clustering of these expression data is shown in Figure 2, with cultivars arranged according to their chilling requirements. In a previous work under our experimental conditions, early cultivars ‘Red Candem’, ‘Flor Red’, ‘May Glo’, ‘86-6’, ‘Precocinho’ and ‘Sunraycer’ required less than 412 chilling hours for dormancy release; intermediate cultivars ‘Carolina’ and ‘Crimson Baby’ needed 412–511 chilling hours; whereas ‘Rose Diamond’ and ‘Big Top’ showed requirements longer than 631 chilling hours [25]. As expected in genes up-regulated after dormancy release, the overall gene expression was higher in early cultivars with low chilling requirements (left) than in late cultivars with higher requirements (right). Interestingly, the peach putative orthologs of *Arabidopsis* genes involved in pollen development programs were mostly grouped in two clusters (I and II), which argues for the existence of evolutionary conserved regulatory circuits orchestrating the coordinated expression of these genes.

**Figure 3:**
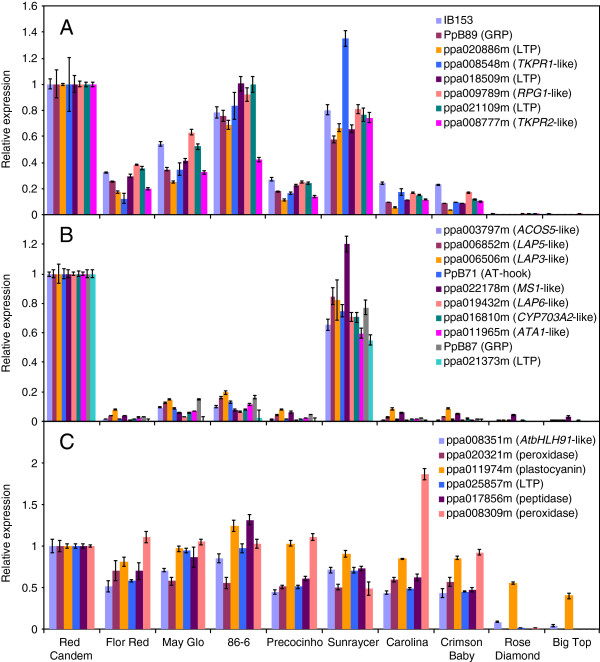
**qRT-PCR of flower-bud late genes in peach cultivars.** The expression of genes from cluster A (**A**), cluster B (**B**) and non-clustered genes (**C**) was measured by qRT-PCR in the RNA samples employed in the microarray hybridization experiment shown in Figure 2. Expression levels are relative to actin. The mean value of expression in ‘Red Candem’ was set to one, and the rest of the values were shown relative to ‘Red Candem’. Data are means from two biological replicates, with error bars representing ± SD.

Quantitative real-time RT-PCR (qRT-PCR) confirmation of microarray hybridization results allowed a more accurate determination of groups of similar expression (Figure 3). Eight genes from the cluster I of Figure 2 were analyzed by qRT-PCR. All of them showed a common pattern, with higher and similar expression values in the cultivars ‘Red Candem’, ‘86-6’ and ‘Sunraycer’, almost undetectable expression in ‘Rose Diamond’ and ‘Big Top’, and intermediate values in the remaining five cultivars (Figure 3A). On the other hand, ten genes analyzed from the cluster II showed a similar expression profile by qRT-PCR, due to their higher transcriptional activity in ‘Red Candem’ and ‘Sunraycer’ (Figure 3B). The gene ppa011974m (plastocyanin-like) from cluster I and other five genes not included in clusters I and II in Figure 2 had a more gradual decline in expression from early to late cultivars (Figure 3C), without drastic differences between cultivars with similar chilling requirements. We employed these qRT-PCR data, based on their improved accuracy over microarray signals, to redefine two clusters of coordinated expression in flower-bud late genes: cluster A including IB153, PpB89 (GRP), ppa020886m (LTP), ppa008548m (*TKPR1*-like), ppa018509m (LTP), ppa009789m (*RPG1*-like), ppa021109m (LTP) and ppa008777m (*TKPR2*-like) (Figure 3A), and cluster B containing ppa003797m (*ACOS5*-like), ppa006852m (*LAP5*-like), ppa006506m (*LAP3*-like), PpB71 (AT-hook), ppa022178m (*MS1*-like), ppa019432m (*LAP6*-like), ppa016810m (*CYP703A2*-like), ppa011965m (*ATA1*-like), PpB87 (GRP) and ppa021373m (LTP) (Figure 3B). The predominant expression in cultivars ‘Red Candem’, ‘Sunraycer’ and to a lesser extent ‘86-6’, indicates an earlier activation of genes involved in microsporogenesis and tapetum development in these cultivars.

### Flower-bud late genes are transiently expressed in anthers

**Figure 4:**
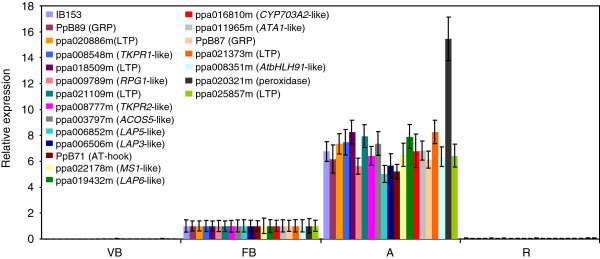
**Tissue-specific expression of flower-bud late genes.** qRT-PCR of flower-bud late genes in vegetative buds (VB), flower buds (FB), anthers (A), and the remaining flower-bud tissue after excising the anthers (R). Buds were collected from ‘Big Top’ cultivar by the middle of February (sample 3 in Figure 5). Expression levels are relative to actin. The mean value of expression in FB was set to one, and the rest of the values were shown relative to FB. Data are means from two biological replicates, with error bars representing ± SD.

The tissue specificity of genes belonging to clusters A and B was studied in the cultivar ‘Big Top’ by qRT-PCR. The transcript accumulation of these genes in vegetative buds was negligible when compared with their expression in flower buds (Figure 4), which precludes a general function of them in dormancy or growth-resumption processes common to both vegetative and reproductive buds. Instead of that, flower-bud late genes from clusters A and B are related to the formation and function of the male gametophyte based on their preferential expression in anthers with respect to other flower-bud tissues (Figure 4). Three transcript models not belonging to clusters A or B, coding for a bHLH transcription factor potentially orthologous to AtbHLH91 from *Arabidopsis* (ppa008351m), a peroxidase similar to At1g44970 product from *Arabidopsis* (ppa020321m), and an LTP family protein (ppa025857m) were similarly expressed in anthers, which indicates that other flower-bud late genes different from those grouped in clusters A and B are also playing a role in anther development processes.

**Figure 5:**
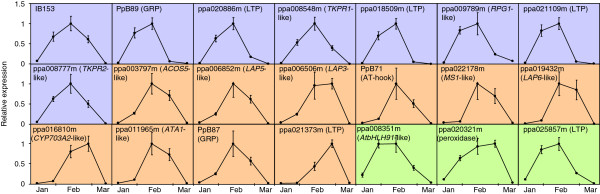
**Temporal expression of flower-bud late genes.** qRT-PCR analysis of flower-bud late genes at five time points from January to March (samples 1–5). Flower buds were collected from ‘Big Top’ cultivar. Genes from cluster A, B and non-clustered genes show violet, brown and green background, respectively. Expression levels are relative to actin. In each graph, the highest expression value was set to one, and the rest of the values were shown relative to this one. Data are means from two biological replicates, with error bars representing ± SD.

The temporal expression of these genes was analyzed in flower buds of ‘Big Top’ collected at different points from the middle of January to the middle of March. Transcriptional expression was induced transiently in genes from clusters A and B, and also in the non-categorized genes ppa008351m (*AtbHLH91*-like), ppa020321m (peroxidase) and ppa025857m (LTP) (Figure 5); but rise and drop of transcript accumulation followed slightly different profiles in the different clusters. Expression of cluster A genes were highly induced in sample 2 (end of January), peaked in sample 3 (middle of February), and started to drop in sample 4 (end of February) to finally reach a low basal level in sample 5, in the middle of March (Figure 5). On the other hand, the induction of cluster B genes in sample 2 was low or absent, and reached a maximum value in sample 3, and in some cases in sample 4. Contrarily to clusters A and B, transcripts belonging to other clusters (simplified in this work as non-clustered genes), such as ppa008351m (*AtbHLH91*-like), ppa020321m (peroxidase) and ppa025857m (LTP) had already a significant expression level in sample 1 (middle of January, Figure 5).

Based on qRT-PCR results shown in Figures 4 and 5, we have determined that flower-bud late genes are transiently expressed in anthers with slight differences in the timing of induction. These results reasonably suggest that cluster-specific differences observed in Figures 2 and 3 are due to differences in the induction time instead of the presence of distinct signals and transduction pathways. Under this hypothesis, cultivar-specific features of clusters A and B and non-clustered genes (Figure 3) could merely describe snapshots of a single transcriptional program taken at different times. Most of cluster B genes are expressed later, leading to cultivar-specific differences at the fixed collection point of 400 chilling hours observed in Figure 3B. On the contrary, earlier non-clustered genes could have acquired a similar maximum expression level at this fixed time in different cultivars (Figure 3C), and A genes could represent an intermediate situation between B and non-clustered genes (Figure 3A). A highly simplified interpretation of these data would suggest the induction of A genes by one or several non-clustered regulatory genes, and the successive expression of B genes induced by a hypothetical transcriptional factor activated or expressed concomitantly with A genes. However a better knowledge on the transcriptional networks affecting tapetum and pollen processes is required to ascertain the plausibility of this hypothesis.

### Flower-bud late genes are expressed during microsporogenesis and pollen maturation processes

**Figure 6:**
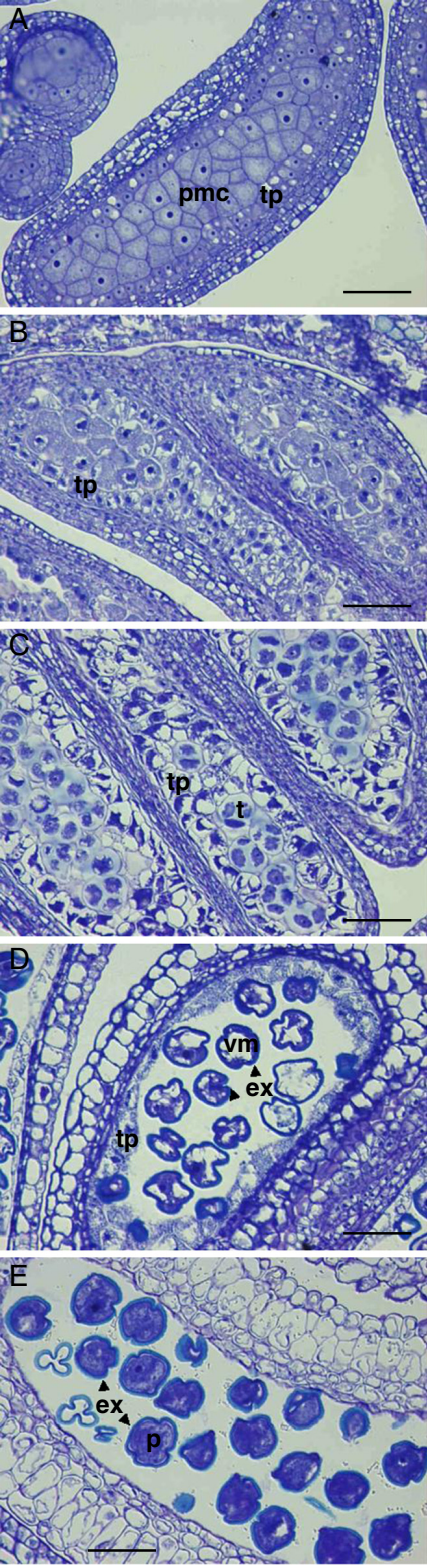
**Light micrographs of anther sections.** Representative anthers from flower buds corresponding to sample 1 (**A**), sample 2 (**B**), sample 3 (**C**), sample 4 (**D**) and sample 5 (**E**) in Figure 5. Scale bars, 50 μm. pmc, pollen mother cell; tp, tapetum; t, tetrad; vm, vacuolated microspore; ex, exine; p, pollen grain.

A histological analysis of anthers on the five samples utilized for qRT-PCR (Figure 5) was performed in order to identify developmental changes associated to the expression of flower-bud late genes. We observed the anthers of three independent buds per sample. In sample 1, fully dormant anthers contained only pollen mother cells and the tapetum layer in a quiescent state (Figure 6A). In sample 2, after 602 chilling hours, flower buds were proximate to dormancy release. At this point, some anthers had already entered microsporogenesis by initiating meiosis of pollen mother cells and tapetum vacuolation (Figure 6B), whereas most of anthers remained inactive. In sample 3, a wide range of developmental stages were observed, from dividing pollen mother cells to isolated microspores, with a high number of anthers showing postmeiotic tetrads surrounded by a callose wall and highly vacuolated tapetal cells (Figure 6C). In sample 4, most of anthers contained vacuolated microspores and a degenerating tapetum (Figure 6D), but one of the buds had also some tetrads. Finally, in sample 5, the tapetum had already disappeared and pollen grains were apparently fully mature (Figure 6E).

Flower-bud late genes were not significantly expressed in samples 1 (dormant buds) and 5 (mature pollen grains), thus they are expected to be involved in one or several processes occurring in samples 2 to 4, as meiotic and mitotic cell division, pollen maturation, synthesis and segregation of substances, and tapetum degeneration. Tapetal cells actively participate in the supply of essential compounds for pollen cells during most of the period covered by these samples and particularly are involved in the synthesis and deposition of sporopollenin; a major component of the pollen cell wall exine. The exine may be identified as a blue light layer surrounding the vacuolated microspores and pollen grains stained in Figures 6D-E, but sporopollenin starts to accumulate earlier, in the tetrad stage [5] (Figure 6C). The temporal expression pattern of flower-bud late genes, peaking in samples 3 and 4 in anthers, in addition to their protein sequence similarity to sporopollenin-related genes of *Arabidopsis*, strongly suggest a role of some of these genes in sporopollenin synthesis and deposition, as detailed below.

### Candidate genes for sporopollenin synthesis and deposition in peach

**Figure 7:**
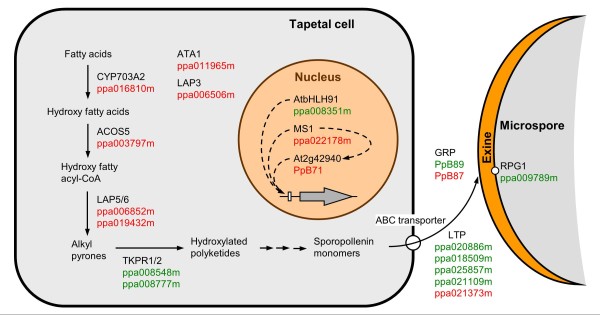
**Schematic view of the sporopollenin synthesis pathway in *Arabidopsis *and peach. ***Arabidopsis* proteins involved in sporopollenin synthesis and deposition, and the transcriptional regulation of late anther development are shown in black. Peach putative orthologs of the genes coding for these proteins are shown in green (cluster A and non-clustered genes with earlier expression) and red (cluster B with later expression). Discontinuous arrows indicate transcriptional interactions. This figure has been adapted from [11].

Those genes having a putative ortholog in the sporopollenin pathway of *Arabidopsis* and others showing LTP or GRP domains (Table 1) have been placed on a schematic picture depicting the hypothetical elements of this pathway in peach (Figure 7). The gene ppa016810m could have a similar role to *CYP703A2* in the hydroxylation of fatty acids [7]. The gene ppa003797m codes for an acyl-CoA synthetase similar to ACOS5, an early and essential function for the synthesis of sporopollenin in *Arabidopsis*[8]. Subsequently, the genes ppa006852m, ppa019432m, ppa008548m and ppa008777m could perform additional steps in the synthesis of sporopollenin monomers similar to the functions exerted by the polyketide synthases LAP5/6 and the tetraketide α-pyrone reductases TKPR1/2 in *Arabidopsis*[9-11]. The resulting sporopollenin monomers are extruded to the locule and deposited on the pollen cell wall with the assistance of LTPs and GRPs [6]. We isolated two GRP-like (PpB87 and PpB89) and five LTP-like genes (ppa020886m, ppa018509m, ppa025857m, ppa021109m and ppa021373m) that could be considered as candidates to perform this role in peach. In addition, ppa009789m gene codes for a protein similar to RPG1 of *Arabidopsis*, a plasma membrane protein involved in exine pattern formation [30]. Two additional flower-bud late genes (ppa011965m and ppa006506m) are respectively putative orthologs of the *ARABIDOPSIS TAPETUM1* gene (*ATA1*), coding for a putative short-chain dehydrogenase/reductase expressed in the tapetum [29] and *LAP3* gene, essential for proper exine formation [27].

The following flower-bud late genes coding for putative DNA-binding and regulatory proteins could be involved in the transcriptional regulation of pollen maturation pathways: ppa008351m, ppa022178m and PpB71 (Figure 7). The *Arabidopsis* potential ortholog of ppa008351m (*AtbHLH91*/At2g31210) codes for a bHLH-type transcription factor that interacts at the protein level with ABORTED MICROSPORES (AMS) and DYSFUNCTIONAL TAPETUM 1 (DYT1), two other bHLH-type factors involved in tapetum development and pollen wall formation [17,34].

On the other side, ppa022178m is the potential peach counterpart of the *Arabidopsis MALE STERILITY1* (*MS1*) gene, which encodes a well-known PHD-domain transcription factor relevant for late tapetum development and pollen wall biosynthesis [15,16,18]. Interestingly, At2g42940 gene, coding for an AT-hook DNA-binding protein highly similar to peach PpB71, was found specifically expressed in the wild-type tapetum after meiosis, and unexpectedly up-regulated in the *ms1* mutant [28]. This prompted to the authors to hypothesize that MS1 was involved in the stage-specific repression of At2g42940 to ensure its expression in a narrow time interval soon after the degeneration of the callose walls surrounding the tetrads. The functional relevance of At2g42940 in pollen cell wall formation was assessed by the generation of RNAi transgenic lines, showing pollen grains with a thinner cell wall, some of which had collapsed [28].

The fact that genes expected to function downstream in the biochemical pathway (TKPRs, LTPs, GRPs) are expressed earlier than the upstream genes seems to be rather inconsistent (Figure 7). However their particular expression profiles do overlap over a certain period of time, suggesting that it could act as a mechanism ensuring the activation of this pathway at the precise time. The complex network of transcriptional and protein interactions between the transcriptional factors involved in early and late anther development in *Arabidopsis*[17,18,34,35] points to an intricate gene regulation pathway. As inferred from the expression studies shown in this work, ppa008351m (bHLH) is expressed earlier than ppa022178m (PHD) and PpB71 (AT-hook) within the regulatory circuits operating in the anther developmental events in peach (Figure 7).

The data presented here constitute an initial genomic approach to unravel anther developmental processes in peach, focusing on sporopollenin synthesis and deposition. In addition, the identification of genes induced during microsporogenesis and pollen maturation processes could assist in the finding of expression biomarkers associated to dormancy release in peach [36].

## Conclusions

This study utilized transcriptomic data from flower buds of peach at different stages of dormancy and several cultivars with different chilling requirements to obtain a list of flower-bud late genes expressed shortly after dormancy release. Some of these genes clustered into two major expression patterns. Their close similarity to genes described in the sporopollenin synthesis pathway in *Arabidopsis* and their transitory expression in anthers coinciding with microsporogenesis events strongly suggests their participation in the biochemical processes required for the formation of the cell wall exine of pollen grains. In addition, three peach regulatory factors with bHLH, PHD and AT-hook domains have been postulated to take part in transcriptional circuits regulating late anther development in peach.

## Methods

### Plant material

The *Prunus persica* [L.] Batsch cv ‘86-6’, ‘Big Top’, ‘Carolina’, ‘Crimson Baby’, ‘Flor Red’, ‘May Glo’, ‘Precocinho’, ‘Red Candem’, ‘Rose Diamond’ and ‘Sunraycer’ were grown in an orchard located at the Instituto Valenciano de Investigaciones Agrarias (IVIA) in Moncada (Spain) under standard agricultural practices. The samples required for qRT-PCR of different cultivars were obtained from flower buds collected after a chilling accumulation of 400 chilling hours (hours below 7°C) [25]. Flower buds of ‘Big Top’ cultivar for microscopy studies and time-dependent expression analysis were collected on the following dates of winter in 2012: 17 January (sample 1, after 460 chilling hours), 30 January (sample 2, after 603 chilling hours), 13 February (sample 3, after 775 chilling hours), 27 February (sample 4, after 936 chilling hours), and 12 March (sample 5, after 1038 chilling hours). Buds for the experiments described in Figure 4 were obtained from sample 3 (see above). Buds were routinely pooled from shoots obtained from three different adult trees.

### Analysis of microarray data

Microarray data utilized in this study are stored in the ArrayExpress database ( http://www.ebi.ac.uk/arrayexpress/) with accession number E-MEXP-3201. We generated a subset of microarray hybridization signals containing only genes and ESTs with higher expression in dormancy-released flower buds (flower-bud late genes) according to previous works [24,25]. The hybridization signal intensity from those ESTs proceeding from the same gene was averaged to have a single hybridization value per gene for each of the ten cultivars used in the experiment. Clustering of gene expression data was performed in the platform Babelomics ( http://babelomics.bioinfo.cipf.es/) [37] using the UPGMA method and the Pearson correlation coefficient as distance.

### Similarity searches

In order to identify putative orthologs of peach flower-bud late genes in *Arabidopsis* we performed a reciprocal blast analysis. First we made a blastp similarity search on *Arabidopsis* database using the predicted translated protein of flower-bud late genes as query. The first hit in the *Arabidopsis* genome was subsequently compared with the peach genome by tblastn search, and those genes found reciprocally by the searches in both the *Arabidopsis* and peach genomes were considered to be putative orthologs (Additional file 1).

### qRT-PCR analysis

Total RNA was isolated from 100 mg of tissue using the RNeasy Plant Mini Kit (Qiagen, Valencia, CA, USA), but adding 1% (w:v) polyvinylpyrrolidone (PVP-40) to the extraction buffer before use. From 1 to 2 μg of total RNA was reverse transcribed with PrimeScript RT reagent kit (Takara Bio, Otsu, Japan) in a total volume of 20 μl. Two microliter of a 20X diluted first-strand cDNA were used for PCR reactions in a final volume of 20 μl. Quantitative real-time PCR was performed on a StepOnePlus Real-Time PCR System (Life Technologies, Carlsbad, CA, USA), using SYBR premix Ex Taq (Tli RNaseH plus) (Takara Bio). Primer pairs are listed in Additional file 2. Cycling protocol consisted of 10 min at 95°C, followed by 40 cycles of 15 s at 95°C for denaturation, and 1 min at 60°C for annealing and extension. Specificity of the PCR reaction was assessed by the presence of a single peak in the dissociation curve after the amplification and through size estimation of the amplified product by agarose electrophoresis. We used as reference a peach actin transcript (ppa007211m) amplified with specific primers. Relative expression was measured by the relative standard curve procedure. Results were the average of two independent biological replicates with 2–3 technical replicates each.

### Light microscopy

Flower buds from ‘Big Top’ cultivar collected at five different dates (samples 1–5, see plant material) were fixed and embedded in London Resin White (London Resin, Woking, Surrey, UK) according to [38]. Sections (about 1 micrometer thick) were cut with a Leica RM2255 microtome (Leica Microsystems, Wetzlar, Germany) using glass knives and fixed to microscope slides. Longitudinal-sections of buds were stained with 0.05% Toluidine Blue O (Merck, Darmstadt, Germany) and examined and photographed with a Leica DM LA microscope (Leica Microsystems).

## Competing interests

The authors declare that they have no competing interests.

## Authors’ contributions

GR participated in the design of the study, performed gene expression studies and drafted the manuscript. FRT carried out the microscopic procedures. CL participated in the design of the study. MLB participated in the design of the study and helped to draft the manuscript. All authors read and approved the final manuscript.

## Supplementary Material

Additional file 1 **Summary of RBA of peach genes showing putative orthologs in the sporopollenin pathway of *Arabidopsis.*** Click here for file

Additional file 2 Primers employed in the qRT-PCR. Click here for file

## References

[B1] Ge X Chang F Ma H Signaling and transcriptional control of reproductive development in Arabidopsis Curr Biol 2010 20 R988 R997 10.1016/j.cub.2010.09.040 21093795

[B2] Berger F Twell D Germline specification and function in plants Annu Rev Plant Biol 2011 62 461 484 10.1146/annurev-arplant-042110-103824 21332359

[B3] Chang F Wang Y Wang S Ma H Molecular control of microsporogenesis in Arabidopsis Curr Opin Plant Biol 2011 14 66 73 10.1016/j.pbi.2010.11.001 21145279

[B4] Piffanelli P Ross JHE Murphy DJ Biogenesis and function of the lipidic structures of pollen grains Sex Plant Reprod 1998 11 65 80 10.1007/s004970050122

[B5] Blackmore S Wortley AH Skvarla JJ Rowley JR Pollen wall development in flowering plants New Phytol 2007 174 483 498 10.1111/j.1469-8137.2007.02060.x 17447905

[B6] Ariizumi T Toriyama K Genetic regulation of sporopollenin synthesis and pollen exine development Annu Rev Plant Biol 2011 62 437 460 10.1146/annurev-arplant-042809-112312 21275644

[B7] Morant M Jorgensen K Schaller H Pinot F Moller BL Werck-Reichhart D Bak S CYP703 is an ancient cytochrome P450 in land plants catalyzing in-chain hydroxylation of lauric acid to provide building blocks for sporopollenin synthesis in pollen Plant Cell 2007 19 1473 1487 10.1105/tpc.106.045948 17496121 PMC1913723

[B8] de Azevedo SC Kim SS Koch S Kienow L Schneider K McKim SM Haughn GW Kombrink E Douglas CJ A novel fatty Acyl-CoA Synthetase is required for pollen development and sporopollenin biosynthesis in Arabidopsis Plant Cell 2009 21 507 525 10.1105/tpc.108.062513 19218397 PMC2660628

[B9] Kim SS Grienenberger E Lallemand B Colpitts CC Kim SY de Azevedo Souza C Geoffroy P Heintz D Krahn D Kaiser M Kombrink E Heitz T Suh DY Legrand M Douglas CJ LAP6/POLYKETIDE SYNTHASE A and LAP5/POLYKETIDE SYNTHASE B encode hydroxyalkyl α-pyrone synthases required for pollen development and sporopollenin biosynthesis in Arabidopsis thaliana Plant Cell 2010 22 4045 4066 10.1105/tpc.110.080028 21193570 PMC3027170

[B10] Dobritsa AA Lei Z Nishikawa S Urbanczyk-Wochniak E Huhman DV Preuss D Sumner LW LAP5 and LAP6 encode anther-specific proteins with similarity to chalcone synthase essential for pollen exine development in Arabidopsis Plant Physiol 2010 153 937 955 10.1104/pp.110.157446 20442277 PMC2899912

[B11] Grienenberger E Kim SS Lallemand B Geoffroy P Heintz D de Azevedo Souza C Heitz T Douglas CJ Legrand M Analysis of TETRAKETIDE α-PYRONE REDUCTASE function in Arabidopsis thaliana reveals a previously unknown, but conserved, biochemical pathway in sporopollenin monomer biosynthesis Plant Cell 2010 22 4067 4083 10.1105/tpc.110.080036 21193572 PMC3027178

[B12] Quilichini TD Friedmann MC Samuels AL Douglas CJ ATP-binding cassette transporter G26 is required for male fertility and pollen exine formation in Arabidopsis Plant Physiol 2010 154 678 690 10.1104/pp.110.161968 20732973 PMC2949020

[B13] Zhang W Sun Y Timofejeva L Chen C Grossniklaus U Ma H Regulation of Arabidopsis tapetum development and function by DYSFUNCTIONAL TAPETUM1 (DYT1) encoding a putative bHLH transcription factor Development 2006 133 3085 3095 10.1242/dev.02463 16831835

[B14] Zhang ZB Zhu J Gao JF Wang C Li H Li H Zhang HQ Zhang S Wang DM Wang QX Huang H Xia HJ Yang ZN Transcription factor AtMYB103 is required for anther development by regulating tapetum development, callose dissolution and exine formation in Arabidopsis Plant J 2007 52 528 538 10.1111/j.1365-313X.2007.03254.x 17727613

[B15] Yang C Vizcay-Barrena G Conner K Wilson ZA MALE STERILITY1 is required for tapetal development and pollen wall biosynthesis Plant Cell 2007 19 3530 3548 10.1105/tpc.107.054981 18032629 PMC2174882

[B16] Ito T Nagata N Yoshiba Y Ohme-Takagi M Ma H Shinozaki K Arabidopsis MALE STERILITY1 encodes a PHD-type transcription factor and regulates pollen and tapetum development Plant Cell 2007 19 3549 3562 10.1105/tpc.107.054536 18032630 PMC2174881

[B17] Xu J Yang C Yuan Z Zhang D Gondwe MY Ding Z Liang W Zhang D Wilson ZA The ABORTED MICROSPORES regulatory network is required for postmeiotic male reproductive development in Arabidopsis thaliana Plant Cell 2010 22 91 107 10.1105/tpc.109.071803 20118226 PMC2828693

[B18] Zhu J Lou Y Xu X Yang ZN A genetic pathway for tapetum development and function in Arabidopsis J Integr Plant Biol 2011 53 892 900 10.1111/j.1744-7909.2011.01078.x 21957980

[B19] Schwalm S Hartmann W Stosser R Pollen development in male fertile and sterile plum cultivars (Prunus-domestica L ssp domestica) Gartenbauwissenschaft 1995 60 145 150

[B20] Werner DJ Creller MA Genetic studies in peach: Inheritance of sweet kernel and male sterility J Amer Soc Hortic Sci 1997 122 215 217

[B21] Lillecrapp AM Wallwork MA Sedgley M Female and male sterility cause low fruit set in a clone of the ‘Trevatt’ variety of apricot (Prunus armeniaca) Sci Hortic 1999 82 255 263 10.1016/S0304-4238(99)00061-8

[B22] Radice S Ontivero M Giordani E Bellini E Anatomical differences on development of fertile and sterile pollen grains of Prunus salicina Lindl Plant Syst Evol 2008 273 63 69 10.1007/s00606-008-0011-5

[B23] Julian C Rodrigo J Herrero M Stamen development and winter dormancy in apricot (Prunus armeniaca) Ann Bot 2011 108 617 625 10.1093/aob/mcr056 21474504 PMC3170150

[B24] Leida C Terol J Martí G Agustí M Llácer G Badenes ML Ríos G Identification of genes associated with bud dormancy release in Prunus persica by suppression subtractive hybridization Tree Physiol 2010 30 655 666 10.1093/treephys/tpq008 20231169

[B25] Leida C Conesa A Llácer G Badenes ML Ríos G Histone modifications and expression of DAM6 gene in peach are modulated during bud dormancy release in a cultivar-dependent manner New Phytol 2012 193 67 80 10.1111/j.1469-8137.2011.03863.x 21899556

[B26] Xing S Zachgo S ROXY1 and ROXY2, two Arabidopsis glutaredoxin genes, are required for anther development Plant J 2008 53 790 801 10.1111/j.1365-313X.2007.03375.x 18036205

[B27] Dobritsa AA Nishikawa S Preuss D Urbanczyk-Wochniak E Sumner LW Hammond A Carlson AL Swanson RJ LAP3, a novel plant protein required for pollen development, is essential for proper exine formation Sex Plant Reprod 2009 22 167 177 10.1007/s00497-009-0101-8 20033437

[B28] Alves-Ferreira M Wellmer F Banhara A Kumar V Riechmann JL Meyerowitz EM Global expression profiling applied to the analysis of Arabidopsis stamen development Plant Physiol 2007 145 747 762 10.1104/pp.107.104422 17905860 PMC2048804

[B29] Lebel-Hardenack S Ye D Koutnikova H Saedler H Grant SR Conserved expression of a TASSELSEED2 homolog in the tapetum of the dioecious Silene latifolia and Arabidopsis thaliana Plant J 1997 12 515 526 10.1046/j.1365-313X.1997.d01-4.x 9351239

[B30] Guan YF Huang XY Zhu J Gao JF Zhang HX Yang ZN RUPTURED POLLEN GRAIN1, a member of the MtN3/saliva gene family, is crucial for exine pattern formation and cell integrity of microspores in Arabidopsis Plant Physiol 2008 147 852 863 10.1104/pp.108.118026 18434608 PMC2409014

[B31] Wilson ZA Morroll SM Dawson J Swarup R Tighe PJ The Arabidopsis MALE STERILITY1 (MS1) gene is a transcriptional regulator of male gametogenesis, with homology to the PHD-finger family of transcription factors Plant J 2001 28 27 39 10.1046/j.1365-313X.2001.01125.x 11696184

[B32] Bielenberg DG Wang Y Li Z Zhebentyayeva T Fan S Reighard GL Scorza R Abbott AG Sequencing and annotation of the evergrowing locus in peach [Prunus persica (L.) Batsch] reveals a cluster of six MADS-box transcription factors as candidate genes for regulation of terminal bud formation Tree Genet Genomics 2008 4 495 507 10.1007/s11295-007-0126-9

[B33] Leida C Conejero A Arbona V Gómez-Cadenas A Llácer G Badenes ML Ríos G Chilling-dependent release of seed and bud dormancy in peach associates to common changes in gene expression PLoS One 2012 7 e35777 10.1371/journal.pone.0035777 22590512 PMC3349671

[B34] Feng B Lu D Ma X Peng Y Sun Y Ning G Ma H Regulation of the Arabidopsis anther transcriptome by DYT1 for pollen development Plant J 2012 72 612 624 10.1111/j.1365-313X.2012.05104.x 22775442

[B35] Ma X Feng B Ma H AMS-dependent and independent regulation of anther transcriptome and comparison with those affected by other Arabidopsis anther genes BMC Plant Biol 2012 12 23 10.1186/1471-2229-12-23 22336428 PMC3305669

[B36] Leida C Romeu JF García-Brunton J Ríos G Badenes ML Gene expression analysis of chilling requirements for flower bud break in peach Plant Breeding 2012 131 329 334 10.1111/j.1439-0523.2011.01946.x

[B37] Medina I Carbonell J Pulido L Madeira S Goetz S Conesa A Tárraga J Pascual-Montano A Nogales-Cadenas R Santoyo J García F Marbà M Montaner D Dopazo J Babelomics: an integrative platform for the analysis of transcriptomics, proteomics and genomic data with advanced functional profiling Nucl Acids Res 2010 38 W210 W213 10.1093/nar/gkq388 20478823 PMC2896184

[B38] Tadeo FR Tudela D Primo-Millo E 1-Aminocyclopropane-1-carboxylic acid-induced ethylene stimulates callus formation by cell enlargement in the cambial region of internodal explants of Citrus Plant Sci 1995 110 113 119 10.1016/0168-9452(95)04173-R

